# Quantitative optimization exploration of the cultivation program for medical product management professionals based on QFD-obstacle integration model

**DOI:** 10.1371/journal.pone.0339633

**Published:** 2026-03-31

**Authors:** Mingming Zhai, Ruochen Feng, Yong Cui, Fang Gao

**Affiliations:** 1 School of Medical Devices, Shenyang Pharmaceutical University, Shenyang, Liaoning, China; 2 School of Foreign Languages and Physical Education, Shenyang Pharmaceutical University, Shenyang, Liaoning, China; PLOS, UNITED KINGDOM OF GREAT BRITAIN AND NORTHERN IRELAND

## Abstract

**Objective:**

The current research on talent cultivation programs for the emerging interdisciplinary field of medical product management in Chinese universities faces the limitation of relying on a single qualitative evaluation, which makes it difficult to meet the growing demand for talent in the industry. This study proposes an innovative framework for the quantitative research on medical product management talent cultivation programs. Using representative domestic universities as empirical cases, it combines qualitative and quantitative methods to assess the quality of talent cultivation programs, laying the foundation for accurately improving the level of medical product management talent training.

**Methods:**

This study utilized methods such as literature review, expert interviews, and questionnaire surveys to explore the cultivation schemes and competency requirements of professional talent in medical product management. Variance analysis and t-tests were employed to analyze differences in talent needs across different social groups. Based on the Obstacle Degree Model, this research analyzed the obstacles affecting market satisfaction with the cultivation of professionals in medical product management. Further, the study developed a QFD (Quality Function Deployment)-Obstacle Integration Model, which combined the importance of demand with the degree of implementation obstacles to quantify and prioritize the core elements of the talent cultivation program.

**Results:**

The study established a cultivation system comprising three dimensions: theoretical teaching, practical teaching, and extracurricular activities, as well as a talent competency requirement system including professional integrity, knowledge reserves, and comprehensive capabilities. Differential analysis revealed discrepancies in the perceptions of talent capabilities between employers and students on certain indices (*P < 0.05*). Satisfaction analysis identified that factors affecting satisfaction were primarily concentrated in the cultivation of professional knowledge and comprehensive skills. Finally, a House of Quality was established, identifying five categories of core elements as the priority improvement directions for the cultivation program (e.g., professional courses, comprehensive practical teaching).

**Conclusions:**

Based on the analysis results, this study proposes optimization strategies for the cultivation program from three aspects: optimizing curriculum design, enriching training models, and strengthening student guidance. This research not only provides a transferable quantitative optimization tool for the cultivation programs of emerging interdisciplinary fields such as medical product management, but also expands the application paradigm of QFD in complex educational systems, advancing the methodological development of this field from a “single demand-driven” approach to a “demand-obstacle dual-driven” one.

## 1 Introduction

The safety and efficacy of medical devices are of overriding importance to human health, and the diversity and innovation are challenging traditional regulatory frameworks. Regulatory authorities worldwide are continuously updating their regulatory systems by integrating regulatory science to ensure and enhance the reliability and effectiveness of medical devices [1,2]. Regulatory science is an advanced interdisciplinary field that has been highly emphasized and developed by the World Health Organization and pharmaceutical powerhouses such as the United States, Europe, and Japan, which has significantly enhanced pharmaceutical innovation and regulatory efficiency [3]. The U.S. Department of Education has officially recognized it as a primary discipline, and has established a comprehensive educational and training system at the undergraduate, master's, and doctoral levels. Europe has also accumulated a wealth of empirical and conceptual academic achievements in regulatory science research and professional talent development, establishing multi-level training institutions and mature models [4,5]. Higher education institutions and professional organizations in Europe systematically train regulatory affairs experts, clinical evaluation managers, and quality assurance specialists who can understand and implement complex regulations through master's programs, advanced training courses, and other formats [6]. In contrast, the development of regulatory science as a discipline and the cultivation of professional talent in China are still in the early exploratory stage, necessitating the adoption of international best practices while aligning with local needs to build a scientific training system.

To enhance the technological, legal, modern, and international levels of drug regulation in China, the National Medical Products Administration (NMPA) launched the “China Drug Regulatory Science Action Plan” in 2019. This plan clearly defines the discipline of regulatory science and emphasizes that the cultivation of high-level professional talent is the foundation and essential guarantee for the sustainable development of regulatory science. In response to national needs, and to address the industry's demand for medical product management professionals as well as the talent shortage faced by regulatory departments, numerous universities across the country have established disciplines in medical device regulatory science. These include pioneering programs such as the Medical Product Management major founded by Shanghai University of Medicine and Health and Shenyang Pharmaceutical University, as well as the medical device regulatory science directions offered by institutions like Shanghai University of Science and Technology and Sichuan University under biomedical engineering. Currently, many graduates have entered the workforce, but the cultivation of talent in medical product management is still in the exploratory stage.

In recent years, domestic scholars have explored the cultivation of medical product management professionals from different perspectives. The research primarily focuses on high-quality talent capability requirements [7–10], factors influencing educational quality and evaluation methods, and curriculum system design [11–13]. The research methods are mainly theoretical and qualitative, with a lack of in-depth empirical research and quantitative analysis. Meanwhile, in the international academic community, particularly in regions with well-established medical device regulatory frameworks (such as the European Union's Medical Device Regulation (MDR) and In Vitro Diagnostic Regulation (IVDR)), the complexity and mandatory nature of the regulatory system have directly led to and shaped systematic empirical research on regulatory science and professional talent cultivation. These studies not only focus on how to build a curriculum system closely aligned with regulatory requirements [14], but also increasingly adopt systematic quantitative or mixed methods to assess training quality and analyze stakeholder needs [14–16]. The “Regulatory Assessor Competency and Training Requirements” document published by the IMDRF GRRP Working Group outlines the competency levels of assessors across multiple dimensions, including regulations, medical device technology, and evaluation procedures. The technical competency framework they established provides the core basis for the subsequent quantitative assessment of technical document reviewers [17,18]. The CORE-MD project team, through large-scale surveys and interviews targeting regulatory agencies, reporting bodies, and clinicians, systematically revealed the core competencies (such as clinical evidence evaluation) and training priorities required by stakeholders in the strict regulatory environment of MDR/IVDR [14,15]. These international empirical studies provide important methodological references for the quantitative analysis of talent cultivation within a clearly defined regulatory framework [19,20]. However, existing research largely focuses on the training of general regulatory affairs professionals in mature and stable regulatory systems. For emerging markets like China, which are in the rapid development phase of regulatory science discipline construction and regulatory frameworks, research on how to systematically design and quantitatively optimize localized talent cultivation programs for a new, interdisciplinary “medical product management” major is still rare. Empirical quantitative analysis of cultivation programs can directly highlight the advantages and shortcomings of existing programs, thus scientifically and objectively guiding the optimization and reform of talent cultivation programs, improving the quality of talent training, and promoting industry development.

To achieve this goal of quantitative analysis, there is an urgent need for a systematic tool that can precisely connect “talent demand” with “training programs.” QFD model perfectly meets this core requirement. This is a quality management method driven by customer (or user) needs, with the core principle being the translation of requirements. By integrating the relationship matrix of “what to do” and “how to do it” into the product design process, it meets customer needs, embodying the quality management principle of focusing on customer satisfaction. Due to its significant advantages in accurately matching customer needs, it has gradually been extended to the service and education sectors [21]. In the realm of talent cultivation, this model has also been widely applied, including in models like the QFD model for enhancing the quality of innovation and entrepreneurship education, and the “Teach, Learn, Do, Use, Create” integrated talent cultivation quality QFD model [21]. The introduction of the QFD model into the quantitative analysis of talent training processes addresses the main contradictions and issues, providing fresh perspectives and methods for enhancing the quality of talent training and expanding the scope of theoretical research in higher education. However, the classic QFD model focuses on the forward conversion from “customer needs” to “technical characteristics,” with the improvement priorities primarily derived from the importance of the needs themselves. It does not systematically integrate the obstacle information derived from satisfaction levels. In the complex system of optimizing educational training programs, the obstacle factors identified through satisfaction surveys directly reveal the key constraints that affect the realization of needs. Evaluating these obstacles is crucial for ensuring the effectiveness of optimization recommendations. Therefore, based on the application of QFD for demand conversion and prioritization, this study innovatively introduces the “Obstacle Degree Model” and constructs a “Demand-Obstacle” dual-dimensional decision framework, which we refer to as the “QFD-Obstacle Integration Model.” This framework first clarifies the initial importance of each need through a demand questionnaire. Subsequently, it identifies the key obstacles affecting social satisfaction and their extent based on a satisfaction survey. Finally, the obstacle degree is used as a correction factor to calibrate the initial importance, thereby generating a comprehensive priority ranking that balances both “need importance” and “realization obstacles.” This integration model not only answers the question of “what should be done,” but also uncovers the key causes of satisfaction constraints, providing a scientifically grounded and implementation-oriented decision basis for formulating optimization recommendations.

To verify the validity and practicality of the aforementioned QFD-Obstacle Integration Model in the emerging educational context of China's Medical Product Management major, and to generate both targeted and universal optimization suggestions for cultivation programs based on this model, this study requires an empirical case that integrates disciplinary pioneering nature, typicality of practical problems and data accessibility. The Medical Product Management major of Shenyang Pharmaceutical University perfectly meets all the above key criteria. As one of the first batch of universities nationwide to be approved for the establishment and enrollment of this major, the challenges it faces in the process of pioneering exploration of its cultivation program, such as demand mismatch and resource constraints, are universally representative at the initial stage of the overall disciplinary construction. Its relatively abundant teaching resources and diversified student groups provide the feasibility for systematically collecting multi-stakeholder data from students, enterprises, regulatory authorities and other parties, thus enabling the full application of the QFD-Obstacle Integration Model. Therefore, this study selects Shenyang Pharmaceutical University as the empirical case, aiming to verify the adaptability of the model with empirical data through an in-depth analysis of its cultivation program and provide a scientific solution for the university to address specific cultivation-related problems. More importantly, it strives to extract systematic quantitative research methods and optimization logic that can be referenced by peer institutions. Specifically, the first step involves multi-faceted survey research to understand the needs and satisfaction levels of regulatory departments, employers, and students regarding the medical product management talent training program, which aims to comprehensively and meticulously understand the specific needs of different groups regarding the talent training programs for medical product management and to precisely gauge their satisfaction with the existing programs. The second step analyzes the impact of various obstacle factors on market satisfaction using the Obstacle Degree Model to systematically identify each obstacle factor and accurately measure the extent of their impact on the market satisfaction with the training of professionals in medical product management. The third step builds a model for enhancing the quality of university medical product management professional talent training based on the “Obstacle Integration Model”, starting from actual social needs, and analyzes the components and mapping relationships of each module, providing a qualitative and quantitative comprehensive evaluation of the importance of each module element. This provides a systematic quantitative research method for enhancing university talent training quality. Ensuring that every aspect and element of the talent training process is subject to precise quantitative analysis and scientifically guided optimization. Finally, based on the analysis results, improvement suggestions are proposed aimed at comprehensively enhancing the quality of training for professionals in medical product management, thus better aligning it with societal development and industry needs.

## 2 Research methods

### 2.1 Indicator system construction

#### 2.1.1 Literature research.

Extensive data is collected from university official websites, CNKI, Web of Science databases, libraries, and other resources on the topic of talent training in medical product management, both domestically and internationally. This includes documents, papers, monographs, and conference materials, which are analyzed for core perspectives and research outcomes concerning theories related to talent training in medical product management. Data is gathered comprehensively from three macro dimensions—professional demeanor, knowledge reserves, and comprehensive abilities—and three micro dimensions—theoretical teaching, practical teaching, and extracurricular activities—to identify potential indicators for the talent capability requirement system and the medical product management professional training scheme system [7,11–13,21–25].

#### 2.1.2 Surveys and expert interviews.

Final indicators are determined through surveys and expert interviews to ensure the accuracy, representativeness, and authority. Survey and interview content are designed based on findings from the literature review, and each indicator's importance is rated on a five-point scale; higher scores indicate greater importance and satisfaction [22]. Interviews and surveys involve regulatory departments for medical device products (regulatory departments), employees of enterprises employing medical product management professionals (enterprises), and students in the medical product management field (students). This process establishes the final evaluation indicator system and determines the weighting coefficients based on each indicator's scores. University heads of medical product management are surveyed to determine the correlation coefficients for capability requirements and training schemes. A preliminary survey sample is first tested, and based on the feedback, modifications are made to the questionnaire to minimize research errors caused by understanding discrepancies and question ambiguities. The scales of surveys are then analyzed for reliability and validity to ensure the effectiveness and reliability of the research.

The study employed an online survey methodology and strictly adhered to ethical guidelines outlined by the relevant institutional review board. The survey did not involve minors, and all data handling was conducted anonymously to ensure participant privacy. Prior to initiating the survey, clear and detailed explanations of the objectives were provided electronically, and digital written informed consent was obtained from all participants (Consent was obtained through WeChat, a widely used digital communication platform). Furthermore, the survey reiterated the purposes of the study and the usage of data to ensure participants were fully informed, thus safeguarding the scientific integrity, reasonableness, and ethical considerations of our research approach. The ethics committee approved our comprehensive consent process.

### 2.2 Differential analysis of professional capability needs among stakeholders

Survey participants are categorized into groups: regulatory departments, enterprises, and students. The importance of element requirements is calculated for each group using SPSS23.0, and variance analysis and t-tests are conducted for differential analysis to investigate if there are differences in needs among these groups. In this study, Analysis of Variance is primarily utilized to test whether the means across multiple populations are equal, which helps to determine if there are significant differences in the importance ratings of various elements needed by different stakeholders. This allows us to assess the overall variance in requirements for elements among various groups. Additionally, the t-test focuses on examining the differences between the means of two populations, employed here to conduct a more detailed comparison between pairs of stakeholders. Through this differential analysis, we particularly address the discrepancies in needs between students and societal actors (regulatory bodies and businesses).

Based on the results of these variance analyses, we can identify specific areas where there is a need to enhance students’ awareness. For instance, if businesses and regulatory bodies place a significantly higher importance on certain elements than students, this indicates a necessity to bolster student understanding of these elements during the educational process. Consequently, we use these analytical insights to revise our talent training programs and schemes. The revision aims to align the skills and knowledge of the trained professionals more closely with actual industry demands. Specifically, we can adjust the curriculum, instructional content, and teaching methodologies based on the information reflected by the differences in needs among various stakeholders. These adjustments are intended to enhance the quality of talent cultivation, ensuring that graduates are better prepared to meet the expectations and requirements of the professional world.

### 2.3 Model for enhancing the quality of talent training in medical product management

#### 2.3.1 QFD theory.

**Fig 1:**
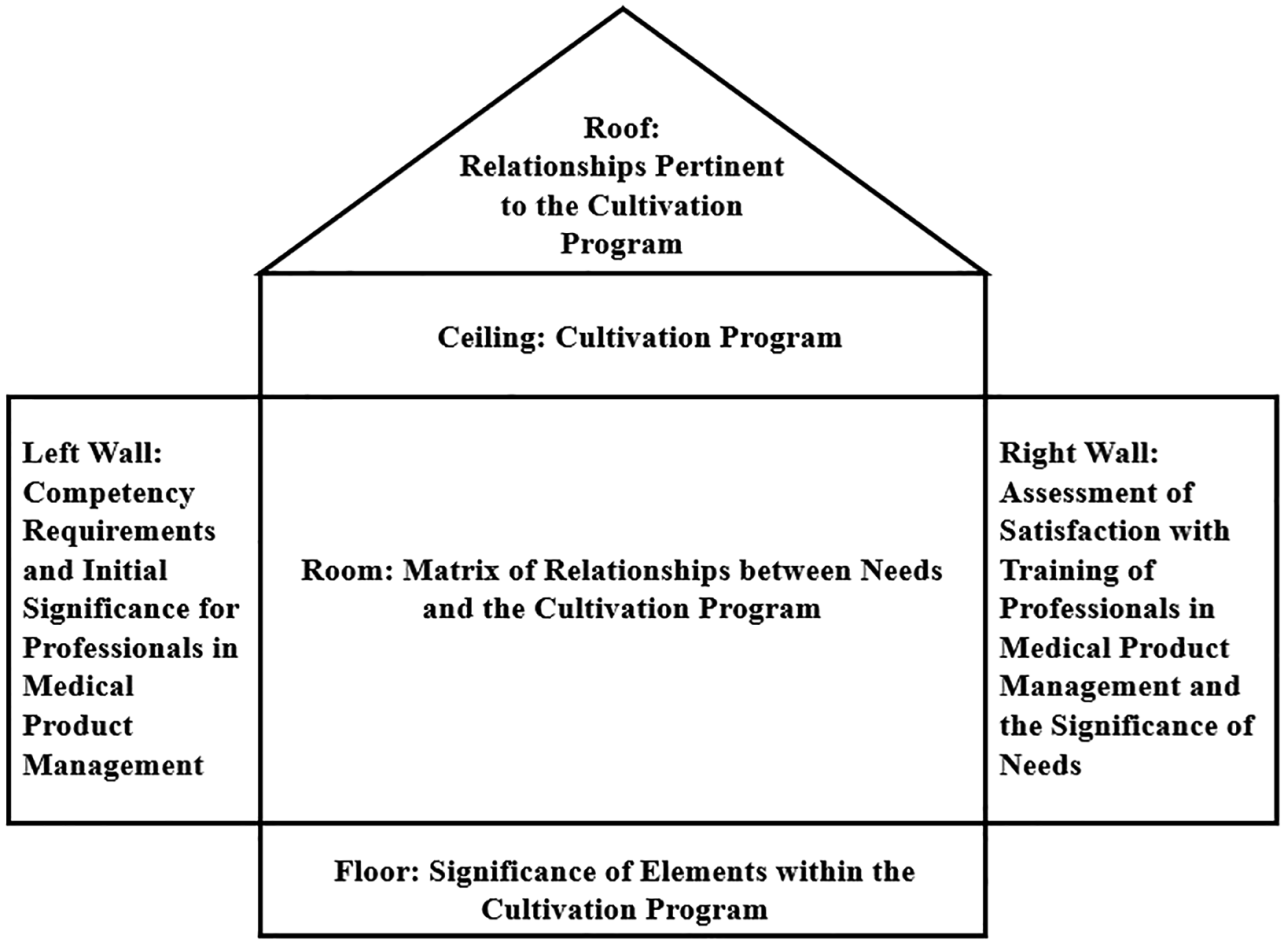
Quality house for enhancing the quality of talent training in medical product management.

This paper employs the QFD method to develop a model for enhancing the quality of medical product management professional cultivation, starting from societal needs for capabilities in medical product management professionals. This demand is translated into the training scheme for medical product management professionals, with the basic structure illustrated in Fig 1. The model studies the interconnections among various components, considers the logical relationships between subsystems and the elements, and establishes a mapping between capability requirements and the training scheme. This process identifies key aspects and elements for enhancing the quality of medical product management professional training, achieving the training objectives. Firstly, the study starts with the societal demand for capabilities in medical product management professionals. It employs a variety of methods including literature review, expert interviews, and surveys to comprehensively and deeply understand these needs, which encompass professional ethics, knowledge reserves, and comprehensive abilities. Subsequently, the study delves into the intrinsic connections between the various components of the model, considering the logical associations between each subsystem and its elements through comprehensive expert surveys. For each dimension of talent capability required, the study meticulously analyzes the corresponding relationships with various elements of the training program. Based on this analysis, a mapping relationship between the talent capabilities needed and the elements of the training program is established. This mapping is not a simple direct correspondence but presents a complex many-to-many association. Certain talent capabilities might closely relate to multiple elements of the training program, and some training elements may impact multiple talent needs. Finally, using the established mapping relationships, the study identifies key components and elements that enhance the quality of training in medical product management. For instance, if the demand analysis reveals a high requirement for risk management and assessment capabilities in professionals, the training program should focus on courses related to these skills. By precisely managing and optimizing these key components, the study transforms societal demands for professional capabilities into a viable talent training program, thus providing a scientific and objective basis for training high-quality, application-oriented professionals in medical product management.

#### 2.3.2 Calculation and methodology of indicator importance: QFD-obstacle integration model.

The importance of each element's indicator is calculated using QFD theory through the following steps:

(1) Calculation of initial importance of capability requirement elements for medical product management professionals, i.e., the demand level. Based on interview and survey results, final indicators are determined and weighted according to the scores given by respondents. The weight of each indicator for all stakeholders equals the proportion of that indicator's importance score relative to the total importance scores. *w*_*j*_ represents the weight coefficient of the indicator, and *dj* represents the importance score of each indicator. The composite weight is the average of the weights across all stakeholders.

$${\text{w}}_{\text{j}}=\frac{{\text{d}}_{\text{j}}}{\sum_{\text{j}=\text{1}}^{\text{m}}{\text{d}}_{\text{j}}},(\text{j}=\text{1},\text{2},\text{3},\cdots ,\text{m})$$ (1)

(2) Assessment of Societal Satisfaction. Based on the results from interviews and surveys, the extent to which each obstacle factor impacts the market satisfaction of professional training in medical product management is determined using the Obstacle Degree Model. A higher obstacle index indicates that the corresponding factor requires prioritized improvement. The formula for calculating the obstacle index is as follows:

$$P_{ij}=\frac{(1-y_{ij})\times w_{j}}{\sum_{j=1}^{n}[(1-y_{ij})\times w_{j}]}\times 100\%$$ (2)

In the formula, *y*_*ij*_ represents the normalized value for the *j*_*th*_ indicator of the *i*_*th*_ evaluation subject, *w*_*j*_ is the weight coefficient of the indicator, and *P*_*ij*_ is the obstacle index of the *j*_*th*_ indicator for the *i*_*th*_ evaluation subject concerning the satisfaction index of training quality in medical product management.
(3) Calculation of Final Importance of Capability Requirement Elements for Medical Product Management Professionals. The initial importance of each factor is adjusted based on its satisfaction scores to yield the final importance and its weight. The formulas are as follows:

Final Importance=Initial Importance×Obstacle Index (3)

Final Importance Weight=Final Importance of Each Requirement/ΣFinal Importance (4)

By integrating the initial importance (demand dimension) and the obstacle index (satisfaction obstacle dimension), the final importance comprehensively reflects the priority of social needs and satisfaction optimization, which is precisely the core of the QFD-Obstacle Integration Model.
(4) Establishment of the “Medical Product Management Professional Capability Requirements— Training Scheme” Relationship Matrix. The relationship matrix is determined by expert scoring, with relationships categorized as strongly related, related, weakly related, and unrelated, with respective scores of 5, 3, 1, and 0 [21].
(5) Calculation of the Importance of Elements in the Training Scheme. To effectively avoid the problems caused by an unreasonable correlation matrix, the importance of each element in the training scheme is calculated using an independent scoring method. The final importance weights of the medical product management professional capability requirements are multiplied by the corresponding degrees of association in the Medical Product Management Professional Capability Requirements—Training Scheme” matrix, and then summed vertically to determine the importance and weight of each element in the training scheme.

## 3 Empirical analysis

### 3.1 Data sources

S University is a provincial comprehensive pharmaceutical university in China, one of the earliest to apply for and receive approval for a specialty in medical product management, officially beginning enrollment in 2019. This paper selects S University as the subject for empirical research to explore the construction and application of a model for enhancing the quality of talent training in medical product management based on QFD theory. Data related to the capability requirements of medical product management professionals, societal satisfaction, and the “Medical Product Management Professional Capability Requirements—Training Scheme” relationship matrix are derived from surveys and interviews. The subjects of these studies include regulatory agencies and employees of enterprises related to the medical product management profession; all students from S University in this specialty since its establishment (classes of 2019–2024) and the heads of the medical product management department at the university. This study on professional capability requirements and societal satisfaction employed stratified sampling, ultimately collecting 270 valid questionnaires. The distribution of the questionnaires is detailed in Table 1, including 25 questionnaires each from freshmen, sophomores, juniors, and seniors, and 50 from graduates; questionnaires from medical device enterprise employees and medical device regulatory departments were filled out by individuals with relevant work experience, most of whom hold at least an undergraduate degree. Expert interviews related to the “Medical Product Management Professional Capability Requirements—Training Scheme” relationship matrix involved seven professional heads responsible for the specialty.

**Table 1:** Distribution of sources for the evaluation scale of medical product management talent training model elements.

Source of Questionnaire	Students	Regulatory Departments	Enterprises	Total
Number of Questionnaires	150	50	50	250

In the reliability analysis, the Cronbachs α coefficients for the capability needs scales and satisfaction evaluation scales of students, enterprises, and regulatory bodies all exceeded the threshold of 0.7. In the validity analysis, apart from the KMO value of the enterprise capability needs scale being 0.62, the remaining KMO values ranged between 0.79 and 0.92. The Bartlett's test of sphericity yielded significant P-values less than 0.05. Factor loadings were also analyzed, with results conforming to the initial hierarchical division. Overall, the survey passed the reliability and validity tests. The training program for medical product management professionals at S University is part of the existing training system [23], detailed in Table 2.

**Table 2:** Training program system for medical product management professionals.

Primary Indicator	Secondary Indicator
Theoretical Teaching P_1_	General Education Courses P_11_
	Subject Foundation Courses P_12_
	Professional Courses P_13_
	Personalized Training P_14_
Practical Teaching P_2_	Basic Practical Teaching P_21_
	Professional Practical Teaching P_22_
	Comprehensive Practical Teaching P_23_
Extracurricular Activities P_3_ (Second Classroom)	Ideological and Political Education P_31_
	Academic Science and Innovation EntrepreneurshipP_32_
	Skills and Qualities Development P_33_
	Social Practice P_34_

### 3.2 Research findings

#### 3.2.1 Construction of the indicator system and calculation of the demand level.

Based on the results of the literature review and survey analysis, the indicator system for the competency requirements of medical product management professionals and the initial weight coefficients (demand level) were established. The indicator system includes three primary indicators: professional integrity, knowledge reserves, and comprehensive capabilities. Under these primary indicators, there are a total of 20 secondary indicators. Specific results are presented in Table 3.

**Table 3:** Competency requirement system for professionals in medical product management.

Primary Indicator	Secondary Indicator	Weight Coefficient			
		Enterprise	Regulatory Department	Student	Comprehensive
Professional Integrity (R_1_)	Professional Ethics R_11_	0.057	0.053	0.050	0.053
	Responsibility R_12_	0.057	0.053	0.050	0.053
Knowledge Reserves (R_2_)	Basic Knowledge of Medical Device Products R_21_	0.053	0.051	0.052	0.052
	Regulatory Basics of Medical Device Products R_22_	0.053	0.051	0.052	0.052
	Knowledge in Related Disciplines such as Medicine, Chemistry Management R_23_	0.050	0.048	0.047	0.048
	Laws, Regulations, and Standards Related to Medical Devices R_24_	0.052	0.051	0.052	0.052
	New Policies and Market Dynamics in Medical Devices R_25_	0.050	0.048	0.051	0.050
	Design and Data Analysis of Clinical Trials for Medical Devices R_26_	0.050	0.045	0.048	0.048
	Understanding of Foreign Regulatory Systems and Policies for Medical Devices R_27_	0.045	0.043	0.049	0.046
	Risk Management and Assessment in Medical Devices R_28_	0.052	0.048	0.051	0.050
	Statistical Methods and Statistical Tools in Regulation R_29_	0.050	0.049	0.048	0.049
	Usage of Modern Tools like Computer Big Data Technology R_20_	0.048	0.045	0.048	0.047
Comprehensive Capabilities (R_3_)	Self-learning and Knowledge Updating R_31_	0.056	0.054	0.051	0.054
	Stress Resistance and Adaptability R_32_	0.055	0.051	0.050	0.052
	Problem Analysis and Solving R_33_	0.055	0.053	0.050	0.053
	Communication and Coordination R_34_	0.054	0.052	0.051	0.052
	Data Analysis and Processing R_35_	0.050	0.049	0.048	0.049
	Team Collaboration R_36_	0.054	0.053	0.049	0.052
	Innovative Thinking and Open Attitude R_37_	0.052	0.050	0.049	0.050
	Risk Assessment and Management R_38_	0.056	0.052	0.050	0.053

As shown in Table 3, the indicator system for the talent capability requirements of the Medical Product Management program covers three core dimensions: professional qualities, knowledge reserve, and comprehensive abilities. The 20 secondary indicators comprehensively encompass the key capabilities required by industry professionals. The weight assignments from the three parties show a consistent overall trend with no significant divergence. Among these, R_31_ (the ability to self-learn and update knowledge) has the highest overall weight (0.054), while R_27_ (understanding of foreign regulatory systems and policies for medical devices) has the lowest overall weight (0.046), with the remaining indicators showing a relatively balanced distribution of overall weights.

#### 3.2.2 Discrepancies in professional competency requirements among different stakeholders.

Using one-way ANOVA, differences in competency requirements for professionals in medical product management among different groups can be assessed. When the p-value of the variance test is less than 0.05, it indicates significant differences between groups. Specific results are shown in Table 4.

**Table 4:** Variance analysis of competency requirement system for professionals in medical product management.

	Secondary Indicators	Variable Values	Variance Test	Welchs Variance Test
Professional Integrity (R_1_)	Professional Ethics R_11_	Enterprise	F = 5.791 P = 0.003***	F = 18.632 P = 0.000***
		Student		
		Regulatory Department		
	Sense of Responsibility R_12_	Enterprise	F = 5.791 P = 0.003***	F = 18.632 P = 0.000***
		Student		
		Regulatory Department		
Knowledge Reserves (R_2_)	Basic Knowledge Related to Medical Device Products R_21_	Enterprise	F = 0.329 P = 0.720	F = 0.392 P = 0.677
		Student		
		Regulatory Department		
	Regulatory Basics of Medical Device Products R_22_	Enterprise	F = 0.757 P = 0.470	F = 0.74 P = 0.480
		Student		
		Regulatory Department		
	Knowledge in Related Disciplines such as Medicine and Chemical Management R_23_	Enterprise	F = 0.072 P = 0.931	F = 0.059 P = 0.942
		Student		
		Regulatory Department		
	Laws, Regulations, and Standards Related to Medical Devices R_24_	Enterprise	F = 2.339 P = 0.098*	F = 1.738 P = 0.182
		Student		
		Regulatory Department		
	New Policies and Market Dynamics in Medical Devices R_25_	Enterprise	F = 4.467 P = 0.012**	F = 3.934 P = 0.023**
		Student		
		Regulatory Department		
	Design and Data Analysis of Clinical Trials for Medical Devices R_26_	Enterprise	F = 4.197 P = 0.016**	F = 3.169 P = 0.047**
		Student		
		Regulatory Department		
	Understanding of Foreign Regulatory Systems and Policies for Medical Devices R_27_	Enterprise	F = 19.49 P = 0.000***	F = 14.515 P = 0.000***
		Student		
		Regulatory Department		
	Risk Management and Assessment in Medical Devices R_28_	Enterprise	F = 3.06 P = 0.048**	F = 2.555 P = 0.084*
		Student		
		Regulatory Department		
	Statistical Methods and Statistical Tools in Regulation R_29_	Enterprise	F = 0.002 P = 0.998	F = 0.002 P = 0.998
		Student		
		Regulatory Department		
	Use of Modern Tools like Computer Big Data Technology R_20_	Enterprise	F = 2.652 P = 0.072*	F = 2.237 P = 0.113
		Student		
		Regulatory Department		
Comprehensive Capabilities(R_3_)	Self-learning and Knowledge Updating R_31_	Enterprise	F = 2.366 P = 0.096*	F = 3.512 P = 0.033**
		Student		
		Regulatory Department		
	Stress Resistance and Adaptability R_32_	Enterprise	F = 1.873 P = 0.156	F = 3.253 P = 0.043**
		Student		
		Regulatory Department		
	Problem Analysis and Solving R_33_	Enterprise	F = 3.484 P = 0.032**	F = 4.597 P = 0.012**
		Student		
		Regulatory Department		
	Communication and Coordination R_34_	Enterprise	F = 0.119 P = 0.888	F = 0.146 P = 0.864
		Student		
		Regulatory Department		
	Data Analysis and Processing R_35_	Enterprise	F = 0.002 P = 0.998	F = 0.002 P = 0.998
		Student		
		Regulatory Department		
	Team Collaboration R_36_	Enterprise	F = 2.605 P = 0.076*	F = 3.44 P = 0.036**
		Student		
		Regulatory Department		
	Innovative Thinking and Open Attitude R_37_	Enterprise	F = 0.849 P = 0.429	F = 1.125 P = 0.329
		Student		
		Regulatory Department		
	Risk Assessment and Management R_38_	Enterprise	F = 2.665 P = 0.071*	F = 4.883 P = 0.009***
		Student		
		Regulatory Department		

***, **, * represent significance levels at 1%, 5%, and 10%, respectively.

**Fig 2:**
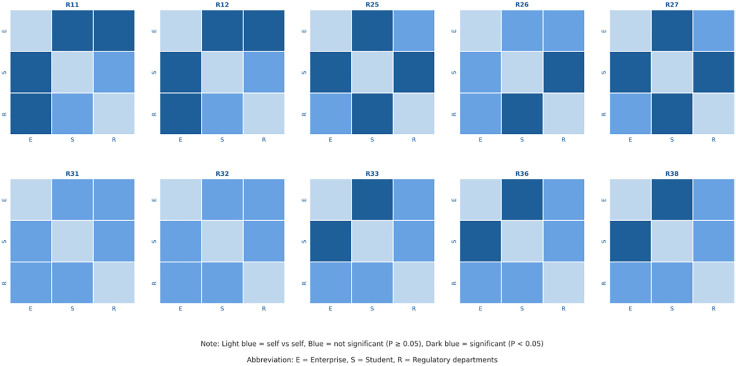
Heatmap of the significance of demand differences between different groups.

Table 4 indicates that significant discrepancies exist between stakeholders in ten areas: Professional Ethics (R_11_), Sense of Responsibility (R_12_), New Policies and Market Dynamics in Medical Devices (R_25_), Design and Data Analysis of Clinical Trials for Medical Devices (R_26_), Understanding of Foreign Regulatory Systems and Policies for Medical Devices (R_27_), Self-learning and Knowledge Updating (R_31_), Stress Resistance and Adaptability (R_32_), Problem Analysis and Solving (R_33_), Team Collaboration (R_36_), and Risk Assessment and Management (R_38_) (*P < 0.05*). For indicators showing significant discrepancies, differences between enterprises and students, enterprises, and regulatory departments, and between regulatory departments and students were analyzed using independent sample T-tests. If the p-value is less than 0.05, it indicates significant discrepancies in the needs for the same indicator between the two stakeholders. Specific results are presented in Fig 2.

Fig 2 reveals the significance of demand differences between pairs of groups. Here, E represents enterprises, S represents students, and R represents regulatory departments. Dark blue indicates significant differences, blue indicates no significant differences, and light blue represents comparisons within the same group. Fig 2 indicates that in terms of Professional Ethics (R_11_) and Responsibility (R_12_) (*P < 0.05*). The demand for these indicators is highest among enterprises, followed by regulatory departments, and lowest among students. Significant discrepancies also exist between enterprises and students in Problem Analysis and Solving (R_33_), Team Collaboration (R_36_), and Risk Assessment and Management (R_38_) (*P < 0.05*), with enterprises having a higher demand than both regulatory departments and students. Significant discrepancies are observed between students and both enterprises and regulatory departments regarding New Policies and Market Dynamics in Medical Devices (R_25_) and Understanding of Foreign Regulatory Systems and Policies for Medical Devices (R_27_) (*P < 0.05*), with students having higher demands than enterprises and regulatory departments. In the area of Design and Data Analysis of Clinical Trials for Medical Devices (R_26_), student demand is significantly higher than that of regulatory departments (*P < 0.05*). No significant discrepancies were found in the comparisons of other indicators.

#### 3.2.3 Diagnosis of obstacles to improving social satisfaction.

**Fig 3:**
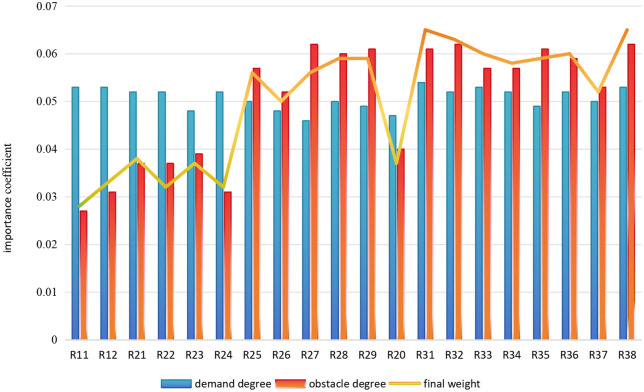
Comparison of the demand level and obstacle level for each indicator.

Building on the competency requirement system for professionals in medical product management, this study uses survey data on the satisfaction levels of various social entities with the current training of professionals in medical products. The obstacle degree model is utilized to further analyze factors impeding satisfaction. Using the SPSS software, the obstacle degrees for each indicator of competency requirements for medical product management professionals are calculated according to the Obstacle degree model's computational method. Based on the base weight coefficients (demand level), the final weight coefficients (the coefficients of both demand level and obstacle level) are calculated. The specific results are shown in Table 5. To visually present the corresponding relationship between the demand level and obstacle level of each indicator, Fig 3 is plotted.

**Table 5:** Satisfaction obstacle factors for talent cultivation in medical product management.

Obstacle Factor	R_11_	R_12_	R_21_	R_22_	R_23_	R_24_	R_25_	R_26_	R_27_	R_28_
Obstacle Degree	0.027	0.031	0.037	0.037	0.039	0.031	0.057	0.052	0.062	0.060
Initial Weight	0.053	0.053	0.052	0.052	0.048	0.052	0.050	0.048	0.046	0.050
Final Weight	0.028	0.033	0.038	0.032	0.037	0.032	0.056	0.050	0.056	0.059
Obstacle Factor	R_29_	R_20_	R_31_	R_32_	R_33_	R_34_	R_35_	R_36_	R_37_	R_38_
Obstacle Degree	0.061	0.040	0.061	0.062	0.057	0.057	0.061	0.059	0.053	0.062
Initial Weight	0.049	0.047	0.054	0.052	0.053	0.052	0.049	0.052	0.05	0.053
Final Weight	0.059	0.037	0.065	0.063	0.060	0.058	0.059	0.060	0.052	0.065

Table 5 clarifies the obstacle levels, initial weights, and final weights for each capability indicator. The core output of the final weight data provides the essential data foundation for the subsequent construction of the Quality Function Deployment (QFD) model.

Fig 3 visually presents the interrelationship between the demand level, satisfaction obstacle level, and final weight of the 20 capability indicators (R_11_-R_38_) in the Medical Product Management program. The blue bar chart represents the demand level (the higher the value, the more the capability is valued by the social groups); the red bar chart represents the obstacle level (the higher the value, the stronger the ability's constraint on market satisfaction); the line represents the integrated priority (calculated by calibrating the demand level and obstacle level, with higher values indicating that the corresponding training aspects need to be prioritized for optimization). The core value lies in precisely identifying the key improvement indicators of “high demand - high obstacle - high weight,” providing an intuitive and scientific decision-making basis for the targeted optimization of the training program. As shown in Fig 3, high obstacle indicators are concentrated in R_27_ (understanding of foreign regulatory systems for medical devices), R_32_ (stress resistance and adaptability), R_38_ (risk assessment and management), R_29_ (statistical methods and statistical tools in regulation), R_31_ (self-learning and knowledge updating), and R_35_ (data analysis and processing). These are the key areas that require breakthroughs in the current training program. Among them, R_38_, R_31_, and R_32_ are core abilities highly valued by society and are also the main constraints on training satisfaction. The final weights for these indicators are ranked highly, making them the top priorities for improvement. R_11_ (professional ethics) and R_12_ (sense of responsibility) have lower obstacle levels, indicating that the satisfaction constraints on the foundational professional qualities in the current training are minimal and do not require significant adjustment.

#### 3.2.4 “Medical product management professional competency requirements—Training program” quality house.

**Fig 4:**
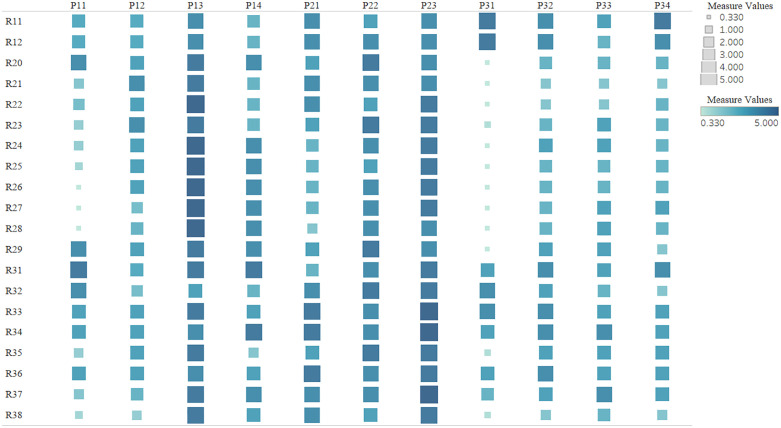
Heatmap of the relationship matrix for the medical product management professional competency requirements—Training program.

A relationship matrix for the “Medical Product Management Professional Competency Requirements—Training Program” was established through the expert scoring method. The relationships are generally classified as strongly related, related, weakly related, and unrelated, with respective values of 5, 3, 1, and 0. The average of the expert scores is used as the correlation score. The importance and weight of each element in the training program are calculated using the independent scoring method, with results presented in Table 6. A heatmap of the quality house is shown in Fig 4. The darker the color and the larger the block, the greater the interrelationship on the horizontal axis.

**Table 6:** Quality house for competency requirement system—Cultivation program in medical product management.

Demand factors	Weight	Theoretical Teaching				Practical Teaching			Extracurricular Activities			
		P_11_	P_12_	P_13_	P_14_	P_21_	P_22_	P_23_	P_31_	P_32_	P_33_	P_34_
R_11_	0.028	2.67	2.67	3.67	2.33	3.67	3.00	3.67	4.33	3.67	3.00	4.33
R_12_	0.033	2.67	2.67	3.67	2.33	3.67	3.67	3.67	4.33	3.67	2.33	3.67
R_21_	0.038	1.67	3.67	4.33	2.33	3.67	3.67	3.67	0.33	1.67	1.67	1.67
R_22_	0.032	2.00	3.00	5.00	2.33	3.67	3.00	4.33	0.33	1.67	1.67	2.33
R_23_	0.037	1.33	3.67	4.33	2.33	3.00	4.33	4.33	0.67	2.33	3.00	2.33
R_24_	0.032	1.33	3.00	5.00	3.67	2.33	3.67	4.33	0.33	3.00	3.00	2.33
R_25_	0.056	1.00	3.00	5.00	3.67	2.33	3.00	4.33	0.33	2.33	2.33	2.33
R_26_	0.050	0.33	3.00	5.00	3.67	2.33	3.67	4.33	0.33	2.33	2.33	2.33
R_27_	0.056	0.33	2.00	5.00	3.67	2.33	3.67	4.33	0.33	2.33	3.00	3.00
R_28_	0.059	0.33	2.33	5.00	3.67	1.67	3.67	3.67	0.33	2.33	3.00	2.33
R_29_	0.059	3.67	3.00	4.33	3.67	3.00	4.33	3.67	0.33	3.00	3.00	1.67
R_20_	0.037	3.67	3.00	4.33	3.67	3.00	4.33	3.67	0.33	2.33	2.33	2.33
R_31_	0.065	4.33	2.67	4.33	4.33	2.33	3.67	4.33	3.00	3.67	3.00	3.67
R_32_	0.063	3.67	2.00	3.00	2.33	3.67	4.33	4.33	3.67	3.00	2.33	1.67
R_33_	0.060	3.00	3.00	4.33	3.00	4.33	3.67	5.00	3.67	3.67	3.00	3.00
R_34_	0.058	3.00	3.00	3.67	4.33	4.33	3.67	5.00	3.00	3.67	3.67	3.00
R_35_	0.059	1.33	3.00	4.33	1.67	3.00	4.33	4.33	0.67	3.00	3.00	3.00
R_36_	0.060	3.00	3.00	3.67	3.00	4.33	3.67	4.33	3.00	3.67	3.00	3.00
R_37_	0.052	1.67	2.33	4.33	3.67	3.67	3.67	5.00	2.33	3.00	3.67	3.00
R_38_	0.065	1.00	1.33	4.33	3.00	3.67	3.00	4.33	0.67	1.67	2.33	1.67
Importance		2.122	2.704	4.315	3.196	3.190	3.713	4.272	1.632	2.823	2.774	2.598
Weight Coefficient		0.064	0.081	0.129	0.096	0.096	0.111	0.128	0.049	0.085	0.083	0.078
Ranking		10	8	1	4	5	3	2	11	6	7	9

As shown in Table 6 and Fig 4, there are differences in the importance of the elements of the training program. The top five elements in terms of importance are as follows: Professional Courses (P_13_), Comprehensive Practical Teaching (P_23_), Professional Practical Teaching (P_22_), Personalized Training (P_14_), and Basic Practical Teaching (P_21_). Among these, three elements belong to the practical teaching category, and two elements belong to the theoretical teaching category. The elements in the extracurricular activities category (P_31_–P_34_) are not among the top five, with relatively lower importance.

## 4 Discussion

### 4.1 Analysis of the differences in professional competency demand recognition among different stakeholders

In review of the established competency requirement system for professionals in medical product management, the evaluation of competencies primarily focuses on three aspects: professional integrity, knowledge reserves, and comprehensive capabilities. As shown in the variance analysis and multiple comparison results in Table 4 and Fig 2, there are significant differences (*P < 0.05*) among the stakeholders on 10 competency indicators. Based on the dimensions of the indicators, the characteristics of these differences can be broken down into the following three aspects:

(1) Concerning professional integrity, enterprises place a higher emphasis on the professional integrity of medical product management professionals compared to regulatory bodies and students. This suggests that for enterprises, professional ethics and a sense of responsibility are essential criteria for talent recruitment. However, students show less concern for these aspects, not recognizing the importance of professional ethics and responsibility in future positions. For students in medical fields, education in professional ethics is crucial as their work directly relates to human life and health. The professional ethics and integrity of practitioners are vital for the stable and healthy development of the medical and health industry. However, the current training process generally emphasizes the cultivation of professional skills over the education in professional ethics [26]. The discrepancies revealed by the analysis reflect the lack of emphasis on professional ethics education in current medical education institutions.
(2) Regarding knowledge reserves, compared to enterprises and regulatory departments, students place greater emphasis on the knowledge reserves of medical product management professionals. This is particularly evident in areas such as New Policies and Market Dynamics in Medical Devices (R_25_), Design and Data Analysis of Clinical Trials for Medical Devices (R_26_), and Understanding of Foreign Regulatory Systems and Policies for Medical Devices (R_27_). This reflects the differences in understanding of competency needs between students and those of enterprises and regulatory bodies. Students focus more on learning about industry policies, systems, and practical knowledge and techniques, believing that the knowledge they acquire is precisely what enterprises and regulatory bodies need and can be directly applied in future work. In contrast, enterprises and regulatory bodies emphasize the industry foundational knowledge that students should possess during their training, such as Basic Knowledge Related to Medical Device Products (R_21_), Regulatory Basics of Medical Device Products (R_22_), and Laws, Regulations, and Standards Related to Medical Devices (R_24_). This discrepancy reveals a cognitive difference between the student group and enterprises and regulatory bodies regarding the competencies required for professionals. Superficially, while students focus more on specialized knowledge and skills, employers prioritize the cultivation of industry foundational knowledge. However, this reflects the emphasis that enterprises and regulatory bodies place on solid foundational knowledge and industry understanding during the talent training process. Medical devices represent a high-tech and rapidly evolving industry, where market dynamics and regulations are constantly changing. Compared to learning specialized knowledge from textbooks, employers place more importance on whether students possess a solid foundation of industry knowledge, sufficient professional awareness, and the capacity for lifelong learning that can adapt to industry developments.
(3) In terms of comprehensive capabilities, enterprises and regulatory bodies place greater emphasis compared to students, particularly evident in areas such as Problem Analysis and Solving (R_33_), Team Collaboration (R_36_), and Risk Assessment and Management (R_38_). This indicates that, compared to students, employers prioritize the cultivation of students’ comprehensive abilities, which aligns with the emphasis seen in knowledge reserves where businesses value students’ lifelong learning abilities to adapt to industry developments. The cognitive discrepancies displayed by students in this area first stem from inadequate guidance in professional recognition, leading to students’ thought patterns not transitioning from academia to industry professionals. Secondly, the proportion of professional practice courses in talent training programs may be insufficient or improperly set, and the lack of industry practice experience among professional course teachers, along with insufficient integration of theory and practice in teaching content, can lead to students undervaluing the cultivation of comprehensive capabilities.

In summary, the fundamental essence of the demand differences among various stakeholders lies in the mismatch between “campus-based standardized training cognition” and “diversified industry employment needs.” This not only reflects the differences in role positioning among different groups but also identifies the “problem targets” for the subsequent judgment of core competency priorities and the optimization of training programs.

### 4.2 Analysis of social demand level and obstacles to satisfaction improvement

Based on the demand level, obstacle level, and final weight data from Table 5 and Fig 3, R_38_, R_31_, and R_32_ are the highest-priority areas for improvement. The core reason for this is their deep connection with industry characteristics:

Risk assessment and management (R_38_) is ranked as the most important due to the critical nature of the medical device industry, which is intrinsically linked to human life and health. Ensuring the safety and efficacy of medical products is crucial, and risk assessment and risk management are effective tools for identifying and mitigating risks, ensuring the safe use of devices, and improving product quality. These processes are integral throughout the product's lifecycle [27]. Therefore, the capability of risk assessment and management is one of the core competencies needed by professionals in medical product management, allowing for an in-depth understanding of potential risks throughout the entire lifecycle of medical devices and enabling the implementation of appropriate risk control measures.

The capability of self-learning and knowledge updating (R_31_) is also critical for professionals in medical product management. The medical device industry is a technology-intensive sector, and with continuous advancements in technology and changing medical needs, new technologies and products are constantly emerging. To keep pace with industry trends and technological iterations, professionals must engage in ongoing learning and continually update their knowledge reserves to ensure they remain at the forefront of the industry. Moreover, the medical device sector is experiencing rapid growth, and related regulations and standards often change. Practitioners need to stay informed about the latest regulations and policies to ensure that products comply with legal requirements. Therefore, the training process for students must equip them with the ability to self-learn and update their knowledge to meet the demands of industry development.

Stress resistance and adaptability (R_32_) are essential capabilities for practitioners in the medical industry. The medical device sector faces multiple pressures including stringent regulatory oversight, intense market competition, continuously evolving technologies, and rapidly changing market demands. Therefore, practitioners need to possess stress resistance and adaptability to manage changes in regulations and address potential issues, ensuring smooth project implementation and sustained development. This capability not only helps in tackling challenges posed by unforeseen circumstances but also drives innovation and improvement.

In summary, the high priority of R_38_, R_31_, and R_32_ is essentially an inevitable requirement for talent capabilities imposed by the medical device industry's characteristics of “rigid compliance, technological iteration, and a multi-pressure environment.” These three types of capabilities are not only core industry demands but also major shortcomings in current training, forming the “core breakthrough points” for the optimization of training programs.

### 4.3 Analysis of results from the “Medical product management professional competency requirements—Training program” quality house

From Table 6 and Fig 4, the relationship between competency training needs and the training program is evident, and the top five elements of importance are identified as follows: Professional Courses (P_13_), Comprehensive Practical Teaching (P_23_), Professional Practice Teaching (P_22_), Personalized Training (P_14_), and Basic Practical Teaching (P_21_).

It is evident that society requires university training programs to focus more on professional courses and various practical courses, ensuring personalized training for students. Professional courses provide students with the necessary industry knowledge and skills, comprising both foundational professional courses and specialized courses. For students in medical product management, foundational professional courses generally include Medical Device Management and Regulations, Clinical Testing Equipment, and Introduction to Medical Devices, aimed at fostering an understanding of medical products and regulatory oversight. Specialized courses include Medical Device Registration Management, Medical Device Production and Distribution Management, and the GMP System for Medical Products, intended to cultivate a deep understanding and application ability in the full lifecycle management of medical devices, equipping students with a solid professional foundation, outstanding practical abilities, and good professional ethics. Additionally, practical courses are an indispensable part of both professional and foundational courses. Basic practical courses in the professional foundation help students master the basic operations of pharmaceutical experiments and the use of experimental equipment. Professional practice courses enable students to apply regulatory knowledge in practical regulatory work, establishing a solid professional foundation and preparing for future work, such as medical device registration, quality management system establishment and maintenance, and risk management. Integrated practical teaching assesses the student's ability to apply and integrate knowledge acquired at university, such as integrated courses in medical product management and capstone projects, allowing students to complete medical device regulatory projects independently or in small groups. Personalized training is facilitated through modular elective courses, allowing students to choose electives based on their interests and career planning and fulfill sufficient credits within the relevant modules. The personalized training program in medical product management includes three major categories: basic general education elective modules, broad category and disciplinary foundation course elective modules, and specialized elective modules in medical product management, aimed at providing students with personalized learning paths to meet their diverse academic interests and developmental needs, thereby cultivating medical product management professionals with unique perspectives and innovative capabilities.

In summary, the core knowledge provided by professional courses, the capability transformation achieved through practical courses, and the demand adaptation realized via personalized training together constitute the core support for talent cultivation in the medical product management major. Their quality directly affects graduates’industry compliance capabilities, practical competence, and career development potential, and is even the key to addressing the previously identified “high-demand and high-obstacle” shortcomings (such as insufficient risk management and self-learning abilities).

## 5 Conclusion and recommendations

This study integrates literature review, expert interviews, and questionnaire surveys to establish a training program system encompassing three dimensions: theoretical teaching, practical teaching, and the extracurricular activities. It also develops a talent capability demand system with three dimensions: professional qualities, knowledge reserve, and comprehensive abilities. Students and employers are regarded as the service targets of talent cultivation, with a thorough investigation into the actual needs of regulatory departments, enterprises, and students. Based on this, variance analysis, obstacle degree model, and Quality Function Deployment (QFD) methods are comprehensively applied. For the first time, the QFD model and obstacle analysis are integrated to form the “Demand-Obstacle” dual-driven “QFD-Obstacle Integration Model,” which is used for the quantitative diagnosis and optimization ranking of the training program. The core research findings and corresponding optimization directions are summarized in Table 7.

**Table 7:** Key findings and optimization directions.

No.	Key Research Findings	Core Issues Revealed by Research Findings	Corresponding Core Optimization Directions
**1**	Structural differences in the demands of multiple social groups (Fig 2): Enterprises, regulatory departments, and students place different emphases on professional integrity, knowledge reserves, and comprehensive abilities.	Reveals the internal differences within the “industry employment standards” and the mismatch between standardized campus training and the diversified market demands.	Implement differentiated guidance and targeted training: Design multiple pathways based on demand differences and strengthen students’industry awareness and career guidance to achieve precise matching of talent supply.
**2**	Based on the comparison of the key competency training priorities within the “Demand-Obstacle” dual-dimensional framework (Fig 3): The cultivation of higher-order abilities, such as self-learning and knowledge updating (R_31_) and risk assessment and management (R_38_), urgently requires optimization.	Exposes the structural shortcomings of traditional curricula in developing higher-order abilities and addressing complex real-world problems.	Enrich the training model: Innovate teaching and practical methods to overcome the bottlenecks in capability development.
**3**	Based on the “Demand-Obstacle” dual-dimensional priority ranking of the training program elements (Table 6): Professional courses (P_13_), comprehensive practice (P_23_), and other elements have the highest weights.	Identifies the “key leverage points” where resource investment and reform measures should be focused, providing a quantitative basis for targeted policy implementation.	Optimize the curriculum design: Focus on core elements and implement systematic, in-depth reforms.

The above diagnostic results validate the feasibility, adaptability, and quantitative effectiveness of the QFD-Obstacle Integration Model. Based on these findings, to precisely enhance the quality of talent cultivation in the Medical Product Management program and better align it with the dynamic demands of the industry and society, this study proposes the following three systematic optimization recommendations.

### 5.1 Optimize curriculum design

In response to the core finding “resource investment and reform measures should focus on key leverage points” identified in Table 7 (Key Finding 3), this study focuses on and systematically optimizes the top five key training elements based on the element priorities output by the Quality Function Deployment (QFD) model (Table 6), in order to most directly improve the alignment of the training program with societal demands. The specific optimization paths are as follows:

#### 5.1.1 Professional curriculum design.

The highest-priority training program element in the House of Quality results is professional courses (P_13_). Within the theoretical teaching (P_1_) category, general education courses (P_11_) and foundational subject courses (P_12_) are ranked 10th and 8th, respectively, while personalized training (P_14_) is ranked 4th. This indicates that for the Medical Product Management program, the most important courses are professional courses. When designing the curriculum, it is essential to focus on personalized training, while general education and foundational subject courses should only meet the basic requirements of the discipline. More class hours should be allocated to developing students’ professional capabilities, enhancing their specialization. Based on student feedback such as “insufficient professional courses” and “lack of management-related knowledge,” along with the high weight of core competencies like R_38_ (risk assessment and management ability), R_28_ (medical device risk management and assessment), and R_29_ (statistical methods and statistical tools in regulation), two aspects need optimization in the curriculum design: First, increase the class hours for core professional courses, with a focus on strengthening courses directly related to R_38_, R_28_, and R_29_, such as medical device registration management, risk management, quality systems, and risk identification tools, addressing the “insufficient professional depth” issue. Second, supplement courses on public management, basic management theory, and other related subjects to solidify the foundation in public management and align with the management attributes of medical product regulation, thereby meeting the post-graduation job requirements of students.

#### 5.1.2 Practical course design.

In the House of Quality analysis results, three practical courses are ranked among the top five in terms of importance, indicating a high demand for practical courses in society. Additionally, an analysis of the open-ended questions in the student survey regarding issues in the curriculum revealed that students generally reported insufficient class hours for practical courses. As a result, they are unable to master the actual processes of the entire medical device lifecycle, such as registration, quality systems, and risk management, and are unable to apply the theoretical knowledge learned to practice. Medical Product Management students, after employment, need to manage the entire lifecycle of medical devices (registration, production, quality, distribution), and skills required for tasks such as preparing registration documents, risk identification, and quality system operation cannot be fully acquired through theoretical teaching alone [28].

Drawing on the “longitudinal practice” model from Harvard Medical School—Cambridge Integrated Internship [29,30], universities should: First, introduce targeted practical courses, such as medical device registration operations, quality system audit practice, and full-process risk management simulations, to cover core job skills. Second, optimize practical teaching faculty by hiring instructors with regulatory or industry experience, or inviting adjunct faculty from enterprises. These instructors can incorporate real industry cases into theoretical classrooms, and through practical courses, enable the conversion from “theory to hands-on practice,” directly enhancing core competencies such as R_38_ and R_33_, and addressing the “disconnect between practice and industry.”

#### 5.1.3 Enhance the professionalism, systematic nature, and scientific approach of courses.

Based on student feedback from the survey (e.g., repetitive content in regulatory courses, layering of computer and regulatory courses) and the requirement in the QFD model results that “professional courses (P_13_) should support multiple core competencies,” the curriculum design must be optimized in three ways to avoid inefficiency: First, addressing faculty shortages through cross-school collaboration: To address the weakness in faculty resources for regulatory science and management courses in medical institutions, we should adopt the “complementary advantages and resource sharing” model from the Nine-School Alliance [31]. This can be achieved by introducing high-quality management courses from other universities through cross-school electives and faculty sharing [32]. Industry experts should also be invited to participate in teaching, sharing real-world cases on compliance management, risk assessment, and other topics, thereby enhancing the professionalism of the courses and aligning them with core knowledge and capability requirements such as R_38_ and R_24_. Second, systematic integration to avoid content duplication: To avoid duplication in regulatory courses, communication between instructors teaching related subjects should be strengthened, and repeated content should be minimized during course preparation. The curriculum system should be more systematic, not limited to individual courses. Instead, it should adopt a comprehensive, multi-angle, and targeted approach to teaching medical device-related regulations. Drawing on the experience of pharmaceutical regulation teaching [33], the course system should be designed with “regulations for the entire lifecycle of medical devices” as the core. The content of regulations, such as the Medical Device Supervision and Administration Regulations, should be split across different courses based on stages such as “registration - production - distribution - use,” avoiding repeated teaching and improving teaching efficiency. Third, adaptation of regulatory tools through interdisciplinary integration: In line with the cultivation goal of “applying regulatory science methods and tools” and the high obstacle level of core competency R_35_ (data analysis and processing), the issue of layering in computer and regulatory courses should be addressed. Drawing on interdisciplinary cultivation models such as Stanford University's Bio-X program [34–36], interdisciplinary courses such as “Regulatory Data Analysis” and “Medical Device Regulatory Information Tools” should be introduced. Regulatory cases (e.g., risk data visualization, compliance process digitalization) should be integrated into computer science teaching, while interdisciplinary faculty should be recruited to ensure students master the “regulation + technology” skills required, aligning with the demands of core competencies like R_35_.

### 5.2 Enrich the training model

To address the two major issues revealed in Table 7, namely, the “mismatch between standardized training and diversified demands” (Key Finding 1) and the “bottleneck in the cultivation of higher-order abilities” (Key Finding 2), it is necessary to implement systematic innovation in the training model, beyond the curriculum, to achieve precise talent development and capability breakthroughs. Specifically, the following two aspects should be advanced collaboratively:

#### 5.2.1 Ensuring personalized training through differentiated cultivation and improved elective course design.

The House of Quality results (Table 6) indicate that personalized training (P_14_) is a core optimization element, and Fig 2 shows significant demand differences between students, enterprises, and regulatory departments. Moreover, the demands of enterprises and regulatory departments differ as well. Students are more focused on R_25_ (new policies and market dynamics for medical devices) and R_27_ (understanding of foreign regulatory systems and policies for medical devices), while enterprises emphasize R_38_ (risk assessment and management) and R_32_ (stress resistance and adaptability), and regulatory departments prioritize R_24_ (laws, regulations, and standards related to medical devices) and R_33_ (problem analysis and solving). This not only reflects the diverse developmental needs of the student group but also demonstrates the differentiated demands of different employers. In light of this dual difference and the high priority of personalized training, differentiated cultivation and flexible elective course design should be implemented to achieve the training goal of “shared foundation, distinctive direction, and personalized adaptation,” while simultaneously aligning with the differing employment standards of enterprises and regulatory departments.

Differentiated cultivation can draw on the “shared foundational courses, distinctive core courses, and elective course selection” model from the pharmacy major [37], and incorporate the broad-based, interdisciplinary course design experience of the MIT EECS department [38]. This approach would involve constructing specialized direction modules, allowing students to select different course combinations based on their chosen direction. Looser elective course rules should be set to increase the proportion of optional courses, meeting students’ interest needs, fully motivating their learning engagement, and implementing a true credit system. This enables students to not only choose their learning direction but also cross-disciplinary electives, broadening their horizons and fostering the development of multidisciplinary talent.

Additionally, leveraging the traditional strengths of Shenyang Pharmaceutical University's pharmacy courses, the personalized elective course system should be optimized as follows: First, break the limits on the number of students per program and major, offering specialized courses such as “Foreign Regulations of Medical Devices” and “Advanced Regulatory Data Analysis” through online courses, video lessons, etc., to meet the diverse needs of students. Second, add elective modules related to the university's strengths (e.g., medical device research and development management in the context of pharmacy), utilizing the university's resource advantages and providing students with differentiated development paths. This ensures that personalized training is deeply aligned with core competency requirements and the university's distinctive characteristics.

#### 5.2.2 Multi-Pronged approach to strengthening core competency development.

Based on the “Demand-Obstacle” dual-dimensional priority comparison of key competency training (Fig 3), core competencies such as R_38_ (risk assessment and management), R_31_ (self-learning and knowledge updating), and R_32_ (stress resistance and adaptability) are among the highest-priority areas for improvement. Enhancing these competencies requires practice in real-world scenarios. The development of such higher-order abilities cannot rely solely on traditional classroom teaching and must be achieved through diverse models such as university-enterprise cooperation, project-based practical training, and industry collaboration. The specific measures are as follows:

(1) Deep Integration of University-Enterprise Collaborative Training to Precisely Align with Industry Needs
University-enterprise cooperation, as a key link between campus training and industry needs, focuses on achieving precise alignment between students’ professional capabilities and the development needs of enterprises through collaborative efforts in talent cultivation, research innovation, and other areas. Common forms of cooperation include internships, joint curriculum development, base construction, and career planning guidance. However, many university-enterprise collaborations still suffer from being superficial and have not effectively addressed the gap between core competency development and the actual demands of the industry.
To address this, we draw on the “integration of education, industry, and research” collaboration model between Johns Hopkins University and its affiliated hospitals. The focus is on precisely empowering the highest-priority core competencies, such as R_38_ (risk assessment and management) and R_32_ (stress resistance and adaptability). Specifically: On one hand, collaborate with medical device companies and regulatory technology institutions to co-build “real-world scenario training bases,” offering projects such as “medical device registration practical training,” “quality system audit practice,” and “full-process risk management simulation.” These projects allow students to deeply engage in the compliance management and risk assessment of real products, directly enhancing R_38_ (risk assessment and management) and addressing the “disconnect between theory and industry.” On the other hand, invite experts with more than five years of regulatory or corporate compliance experience to serve as mentors, co-teaching alongside faculty. Through specialized courses on topics like “handling compliance crises” and “cross-department collaboration case studies,” industry practical experience is shared, directly aligning with the enterprise's employment standards, and enhancing competencies such as R_32_ (stress resistance and adaptability) and R_33_ (problem analysis and solving).
(2) Implement Core Competency-Oriented Project-Based Teaching to Strengthen Practical Application
To address the issues of fragmented competency development and the lack of real-world scenario-driven learning in traditional teaching, and in response to the high obstacle levels of R_31_ (self-learning and knowledge updating) and R_33_ (problem analysis and solving), a comprehensive project on “Medical Device Lifecycle Management” is designed. In this project, students work in groups to fully participate in the entire process from product registration and quality system establishment to risk assessment.
The project is deeply integrated with real-time industry policies (such as updates to the EU MDR/IVDR, and new domestic medical device regulatory regulations), compelling students to actively focus on R_25_ (new policies and market dynamics for medical devices) and R_27_ (understanding of foreign regulatory systems and policies for medical devices), thereby enhancing their self-learning ability through policy tracking and adaptation. At the same time, the project implementation intentionally sets up “compliance risk scenarios” (e.g., product inspection failures, adapting processes after regulatory updates), allowing students to develop their stress resistance and adaptability (R_32_) in simulated real-world situations. This approach directly matches the core industry need for the ability to handle complex scenarios.
(3) Leverage Industry Resources to Build Competency Verification Platforms and Assess Training Effectiveness
To strengthen the practical application and evaluation of core competencies, a diversified competency verification platform is established by linking industry resources, such as enterprises and regulatory departments. On one hand, an “Medical Device Regulatory Skills Competition” is organized, focusing on core indicators like R_38_ (risk assessment and management) and R_35_ (data analysis and processing). Practical components, such as “quality system defect identification,” “risk assessment report writing,” and “regulatory data visualization analysis,” are set up, with compliance experts from enterprises and regulatory department staff serving as judges. This competition aims to promote learning through the competition and encourage improvements through the evaluation, providing a direct test of students mastery of core competencies. On the other hand, regular “Regulatory Case Discussion Seminars” are held, where real-world medical device compliance disputes and risk events from the industry (e.g., product adverse event handling, corrective actions for illegal production) are collected. Students are divided into groups to discuss and present solutions. This case breakdown strengthens R_33_ (problem analysis and solving) while also allowing students to gain a direct understanding of the industry's application requirements for core competencies, narrowing the gap between campus training and industry needs.

### 5.3 Strengthen student guidance

In response to the “significant cognitive differences between students and employers regarding professional qualities, comprehensive abilities, etc.” revealed in Table 7 (Key Finding 1), and considering the issues some students face with unclear professional awareness and uncertain career planning, it is necessary to implement guidance throughout the entire university journey. This will help students understand industry demands and career development paths, narrowing the gap between “campus perception” and “workplace standards.” The guidance work should focus on the following two dimensions:

#### 5.3.1 Increase students’ professional awareness and professional identity.

As Medical Product Management is an emerging field, some students enter the program with unclear perceptions and random choices, leading to low professional identity, confusion about future career planning, and lack of motivation to study [39]. This indirectly affects the development of R_31_ (self-learning and knowledge updating). Therefore, guidance should focus on systematically enhancing students’professional awareness, professional identity, and sense of career belonging. Specifically, the following actions should be taken: First, increase awareness of the major: Efforts should be made to promote the major and raise its social recognition, encouraging more students to proactively choose this field rather than making random or uninformed choices. This can be achieved by advertising the demand for medical product management professionals in industries such as enterprises and regulatory departments.

Publicizing through internship opportunities in enterprises can increase the social recognition of the major and create more job opportunities. Second, implement professional awareness education: Introduce courses such as a compulsory introduction to the major for freshmen, professional awareness education activities, and integrate professional awareness into career planning courses [39]. This will allow students to understand the content of their chosen field and its future development, thus enhancing their professional awareness and identity. Drawing on the Oxford University mentorship system, which is a highly effective method for cultivating professional awareness and identity, each student is assigned a professional mentor who guides their academic research, as well as personal development and career planning [40]. Through in-depth communication and collaboration with their mentors, students can better understand the essence and value of their field, leading to a strong professional identity [41]. Universities may also consider introducing a similar mentorship system in the Medical Product Management program to enhance students’ professional awareness and identity.

#### 5.3.2 Enhance the development of students’ professional integrity and abilities.

Fig 2 shows that enterprises’ demand for R_11_ (professional ethics) and R_36_ (team collaboration) is significantly higher than that of students (*P < 0.05*). Additionally, as Medical Product Management involves human health, professional qualities (such as professional ethics and responsibility) form the foundation for core competencies like R_38_ (risk assessment and management) and R_36_ (team collaboration)—lacking professional integrity can directly affect the rigor of risk control and the coordination of teams. Therefore, the cultivation of professional qualities should closely align with industry demands and core competencies. Specifically, the following actions should be taken: First, strengthen professional ethics education: Drawing on Stanford University's interdisciplinary social ethics teaching model [42,43], offer courses in medical ethics. Through the analysis of real-world cases, such as illegal medical device production or compliance risk management, students should understand the crucial role that professional ethics play in job responsibilities and cultivate a respect for life and a commitment to compliance. Second, integrate ability cultivation into practical scenarios: Use methods such as “team collaboration on regulatory simulation projects” and “cross-group debates on compliance cases” to cultivate students’communication and coordination abilities (R_34_) and problem analysis and solving abilities (R_33_). Integrate professional quality requirements into course assessments. For example, in risk management courses, “compliance awareness” should be used as one of the evaluation criteria to ensure that professional qualities and core competencies are developed in parallel.

In summary, to enhance the quality of talent cultivation in the Medical Product Management program, the cultivation program should be proactively optimized across three key dimensions: optimizing course settings, enriching the training model, and strengthening student guidance. Optimizing course settings aims to equip students with necessary professional skills, evidenced by assessment scores and competition performances, which indicate the effectiveness of the curriculum. Enriching the training model through varied paths and industry collaborations enhances practical skills and industry knowledge, impacting competencies in teamwork and communication. Additionally, improving professional awareness and vocational skills prepares students for professional challenges and aligns with employer expectations concerning professional ethics and innovation.

Establishing a robust assessment system is essential for maintaining training quality. This system should evaluate the effectiveness of course settings, practical teaching, student competencies, and employer feedback. These assessments support the effective implementation and continual optimization of the training program, ultimately contributing to the medical device industry by providing well-prepared, application-oriented professionals.

## 6 Research implications and outlook

The growing societal demand for professionals in medical device regulation in China, coupled with the current limited supply of such talent, is becoming increasingly evident. As a nascent interdisciplinary field, the talent cultivation system for medical product management is still under exploration and development. Existing research in this area mainly relies on qualitative analysis, lacking quantitative optimization methods tailored to the unique characteristics of this emerging discipline. The core value of this study lies in the innovative construction of the “QFD-Obstacle Integration Model,” which combines the demand-driven logic of classic QFD with the obstacle identification functionality of the obstacle degree model. This approach overcomes the limitation of traditional QFD, which focuses solely on “demand importance” while neglecting “implementation obstacles,” providing a two-dimensional quantitative optimization framework for emerging disciplines based on both “demand” and “obstacles.” This method is particularly well-suited to fields like medical product management, where the discipline system is underdeveloped and there is a mismatch between demand and talent cultivation. It not only addresses the issues of insufficient empirical support and quantitative analysis in existing studies but also offers a scientifically sound and practically actionable basis for optimizing training programs by accurately identifying core indicators of “high demand - high obstacles.” This innovation not only provides a portable quantitative optimization tool for training programs in emerging interdisciplinary fields like medical product management but also extends the application paradigm of QFD in complex educational systems, driving the development of the field from a “single demand-driven” to a “dual demand-obstacle-driven” methodology.

From a practical perspective, the talent capability demand system, group difference analysis results, and quality function deployment (QFD) optimization ranking established in this study provide systematic guidance for higher education institutions in talent cultivation. It not only clarifies the prioritization of core elements such as professional courses and practical teaching but also offers three targeted suggestions for course optimization, model innovation, and student guidance. These recommendations help universities enhance the professionalism and specificity of their talent training, ensuring a better alignment between industry needs and campus-based education. Furthermore, this model provides a quantitative logic that can be referenced for optimizing training programs in other emerging disciplines related to regulatory sciences.

Although this study, through a rigorous mixed-methods design and the construction of the “QFD-Obstacle Integration Model,” has achieved a quantitative diagnosis and optimization of the medical product management program, certain limitations still exist. First, in terms of data collection, the empirical data in this study are primarily based on Shenyang Pharmaceutical University as a typical case. While this validates the scientific nature and practical feasibility of the “QFD-Obstacle Integration Model,” the singular institutional attribute of the sample limits the external validity and transferability of some optimization recommendations. Second, this study mainly focuses on the analysis and optimization of the current situation, with relatively insufficient predictive research on the future changes in talent demand driven by the rapid development of the industry. This may affect the adaptability of the research findings in long-term planning. Based on these limitations, future research can be further deepened in three ways: first, expanding the empirical breadth by incorporating data from universities at different geographic locations and levels, as well as from a broader range of employers, to enhance the generalizability of the research conclusions; second, introducing a dynamic perspective by combining policy analysis and technology forecasting methods to build a dynamic talent demand prediction model, improving the foresight and resilience of training programs; third, promoting the evolution and transfer of the model, exploring its integration with big data and machine learning technologies to optimize computational efficiency, and extending the validated framework to related fields in regulatory science, such as pharmaceuticals and cosmetics. This would provide stronger methodological support for the construction of a future-oriented high-level regulatory professional talent training system.

In conclusion, this study provides a new path for the systematic and precise optimization of talent cultivation in medical product management. Through continued advancements in data, forecasting, and technology, this model and its related findings are expected to offer solid support for the construction of a more resilient and forward-looking regulatory science talent cultivation system in China. This, in turn, will empower the high-quality development of the medical device industry and the comprehensive enhancement of regulatory effectiveness.

## Supporting information

S1 File Original data. (ZIP)
