# Supplementary material for: Quantitative optimization exploration of the cultivation program for medical product management professionals based on QFD-obstacle integration model
Source: PLoS One. 2026 Mar 31;21(3):e0339633. doi: 10.1371/journal.pone.0339633 (PMC13038009; doi:10.1371/journal.pone.0339633)
Supplement: S1 File — (ZIP) [file pone.0339633.s001.zip › original data/Data Description.docx]

This compressed file contains the following four sets of research data used in the study:
(1) Evaluation Scale of Elements in the Training Model for Medical Product Management Talents - Enterprises: Questionnaire results collecting data from enterprise professionals on key elements of the training model.
(2) Evaluation Scale of Elements in the Training Model for Medical Product Management Talents - Regulatory Authorities: Questionnaire results collecting data from regulatory agency professionals on key elements of the training model.
(3) Evaluation Scale of Elements in the Training Model for Medical Product Management Talents - Students_150_150: Questionnaire results collecting data from students on key elements of the training model.

(4) Research on the Correlation between the Elements of Competency Requirements for Talents in the Medical Product Management Major and the Training Program_7_7(1): Survey results on the correlation between competency requirements and the training program.
